# Thyroid Stem Cells But Not Differentiated Thyrocytes Are Sensitive to Slightly Increased Concentrations of Heavy Metals

**DOI:** 10.3389/fendo.2021.652675

**Published:** 2021-04-19

**Authors:** Fiorenza Gianì, Roberta Masto, Maria Antonietta Trovato, Annarita Franco, Giuseppe Pandini, Riccardo Vigneri

**Affiliations:** ^1^ Endocrinology, Department of Clinical and Experimental Medicine, Garibaldi-Nesima Medical Center, Catania, Italy; ^2^ Surgical Oncology, Garibaldi-Nesima Medical Center, Catania, Italy; ^3^ Crystallography Institute, National Research Council, CNR Catania Section, Catania, Italy

**Keywords:** environment, heavy metals, thyroid stem cells, thyroid, thyrospheres

## Abstract

Thyroid cancer incidence is markedly increased in volcanic areas where residents are biocontaminated by chronic lifelong exposure to slightly increased metals in the environment. Metals can influence the biology of living cells by a variety of mechanisms, depending not only on the dose and length of exposure but also on the type and stage of differentiation of target cells. We explored the effect of five heavy metals (Cu, Hg, Pd, W and Zn) at nanomolar concentrations (the biocontamination level in residents of the volcanic area in Sicily where thyroid cancer is increased) on stimulating the proliferation of undifferentiated (thyrospheres) and differentiated human thyroid cells. Thyrosphere proliferation was significantly increased after exposure to each individual metal and a greater stimulating effect was observed when a mixture of the examined metals was used. No effect was seen in differentiated thyrocytes. For all metals, the dose-response curve followed a biphasic pattern that is typical of hormesis. Thyrosphere growth concerned the size rather than number, except with the metal mixture. An altered morphology was also observed in metal-treated thyrospheres. Metal-induced proliferation was due to activation of the ERK1/2 pathway, as confirmed by growth inhibition when ERK1/2 signaling was blocked. These studies show that stem/precursor thyroid cells are sensitive to small increases in environmental metal concentrations that are harmless for differentiated thyrocytes.

## Introduction

A dramatic worldwide increase in thyroid cancer has been observed in recent decades, despite the incidence of most other cancers having remained stable or only increased slightly during the same period (1).

Indirect but strong evidence supports the possibility that this increase is not only “apparent” (due to the identification of a large number of small tumors that clinically are not relevant) (2, 3), but that a “true” increase in number and a change in thyroid cancer aggressiveness is also occurring (4, 5). These considerations imply that environmental factors are promoting the initiation and progression of thyroid cancer: radiation, and dietary and atmospheric pollutants related to the industrialized way of life are the most likely factors.

Metals are natural components of the Earth’s crust. These inorganic elements can play an important role in human biology, both as essential nutrients and also as potentially toxic compounds. At abnormal concentrations, metals may have detrimental effects on a variety of cell functions, including growth, transformation and survival. These toxic effects may be exerted by different metals at different concentrations in different cell types, depending on the specific cell biology and intracellular metal accumulation and metabolism. In the last few decades, the industrialized lifestyle has involved a progressively greater use of metals and the consequent environmental pollution, raising some concerns for the potential toxic effects of human bio-contamination due to chronic exposure to increased metals in the environment (6).

We recently reported that the incidence of thyroid cancer is double in the Mt. Etna volcanic area of Sicily relative to adjacent non-volcanic areas (7), an observation that reflects previous observations in other volcanic areas (2, 3, 8–13). In the Sicilian volcanic area, a significant non-anthropogenic heavy metal pollution is present and causes the bio-contamination of residents (14), suggesting the possibility of a cause-effect relationship with the increased incidence of thyroid cancer. However, in the urine of volcanic area residents, only boron, molybdenum, palladium and tungsten are at higher levels than the 95^th^ percentile of Italian standard values (14), posing the question of whether chronic exposure to a small increase in environment metals, often within the “normal” limits for each single metal, can have a detrimental effect on thyroid cell biology.

A detrimental effect of metals at slightly increased concentrations is possible, as indicated by the biological effects of many metals at very low concentrations, activating a biphasic, non-linear hormetic response in exposed cells (15). Moreover, a combined effect of different metals at only slightly increased levels but acting synergistically is also possible. Finally, another possibility is that cells at a lower level of differentiation, such as embryonic cells, may be more sensitive to the detrimental effects of metals (16). We recently documented that tungsten, at concentrations in the range of values measured in the urine of residents from the volcanic area, promoted proliferation, inhibited apoptosis and induced the characteristics of a transformed phenotype in human thyroid stem/precursor cells but not in differentiated thyrocytes (17). These data indicate that a small increase in tungsten in the culture medium, which is harmless for differentiated thyrocytes, can affect the biology of stem/precursor thyroid cells, inducing the characteristics of pre-neoplastic transformation.

The present study is aimed at investigating whether five heavy metals that are slightly increased in the volcanic environment and their mixture differently affect human thyroid cells at different stages of differentiation.

To this end we used human mature thyrocytes and undifferentiated thyrocytes in the form of thyrospheres, aggregates of thyroid stem cells and precursors of thyrocytes at different level of differentiation. Thyrospheres are able to either differentiate in mature thyrocytes or produce additional thyrospheres (by a process named self-renewal) depending on the culture medium used.

## Materials and Methods

### Investigated Heavy Metals

The following metals were investigated in the chemical form of salt compounds: copper (Cu) used as CuSO_4_, zinc (Zn) as ZnCl_2_, mercury (Hg) as HgCl_2_, palladium (Pd) as PdCl_2_ and tungsten (W) as Na_2_WO_4_. All of these salts were obtained from Sigma-Aldrich (St. Louis, MO, USA). CuSO_4_ and Na_2_WO_4_ were dissolved in deionized water, ZnCl_2_ and PdCl_2_ were dissolved in deionized water containing 10% 1N HCl and HgCl_2_ was dissolved in absolute ethanol.

Stock solutions were prepared for all of these salts at 10 mM concentrations and stored at 4°C until used, when they were further diluted to the indicated concentrations in RPMI medium containing 0.1% BSA.

### Cultures of Human Thyroid Cells

Normal human thyroid tissue specimens were obtained from 14 euthyroid female patients aged 30–65 years who had undergone surgery (Oncology Surgery unit of the Garibaldi-Nesima Medical Center in Catania) for a solitary thyroid nodule classified TIR-3 at cytology which was shown to be benign at pathological examination. Written informed consent was obtained in all cases and the study was approved by the local Ethics Committee (n.12/2015/CECT2).

The isolation and culture of human thyroid cells were performed as previously reported (17). Briefly, after the meticulous removal of fibrous tissue, the just excised fresh thyroid tissue was first minced with sterile scissors and then digested with collagenase IV (1 mg/ml, Sigma-Aldrich) for 2 h at 37°C. The resulting cell suspension containing intact and fragmented thyroid follicles was centrifuged (400 *g* for 10 min), and the pellet was suspended in RPMI 1640 culture medium (Sigma) supplemented with 2 mM glutamine (Sigma), 2.5% heat-inactivated fetal bovine serum (FBS, from Invitrogen), B-27 (1:100, Thermo), insulin-transferrin-sodium selenite liquid medium supplement (ITS, 1:200, Thermo), and epidermal growth factor (EGF, 1 ng/ml; Sigma), before being incubated at 37°C in a 5% CO_2_ atmosphere. After 12–24 h, viable thyroid cells attached to the flasks and supernatants containing unattached cells were transferred into fresh flasks, and the cells were cultured at 100% confluence. Residual fibroblasts, when present, were depleted using magnetic anti-fibroblast beads (Miltenyi Biotec) according to the manufacturer’s instructions.

Under these conditions, follicular cell monolayers are formed after 1–2 days. The culture medium was then renewed every 2–3 days and all experiments were performed with cells at passages 3–7.

Differentiated thyrocytes in primary culture were grown in the above descrived RPMI medium supplemented with 1 mU/ml bovine thyroid-stimulating hormone (TSH) (Sigma) and deprived of EGF.

Stem/progenitor thyroid cells were obtained by the trypsinization of primary culture thyroid cells and seeding cells at a density of 1–5 × 10^4^ cells/ml. These were cultured in ultralow attachment plastic flasks (Eppendorf) in the standard stem cell medium, RPMI 1640 supplemented with 2% B-27 (Thermo) enriched with EGF (20 ng/ml, from Sigma). Under these conditions, stem/progenitor thyroid cells form free-floating thyrospheres, aggregates of thyroid stem cells and precursors of thyrocytes at different levels of differentiation. Human thyrospheres generated with these procedure were characterized for phenotypic and genetic markers as previously reported (18).

### Cell Proliferation Measurement

Cell proliferation was evaluated by 5-bromo-2’deoxyuridine (BrdU) incorporation. Thyrocytes and precursor/stem cells disaggregated from thyrospheres were seeded at a cell density of 10,000–15,000 cells/well in a 96-well microtiter plate in phenol red-free RPMI medium supplemented with 2% FBS. After adhesion and overnight starvation in RPMI medium with 0.1% BSA, heavy metals were added to the culture medium at the indicated concentrations. After 48 hours of exposure to metals, cells were labeled with BrdU (DELFIA cell proliferation kit, PerkinElmer) for an additional 24 hours and BrdU incorporation was evaluated according to the manufacturer’s instructions.

To further evaluate the metal effect on thyroid stem/progenitor cell growth, we also measured thyrosphere numbers and volume after exposure to metal. Thyrospheres were first dissociated mechanically and enzymatically into single cells and then seeded at a cell density of 3000 cells/well in a 96-well microtiter plate in phenol red-free RPMI medium supplemented with 0.1% B-27, before being exposed for 8–10 days to metals at the concentration causing the greatest effect at BrdU incorporation. The secondary thyrosphere number was counted in each well (96-well plate, 100 μL) and the average of four wells was averaged for each condition. Phase-contrast images of morphological changes of secondary thyrosphere formation were captured by an Olympus optical microscope supported by a DP20-5E digital camera. Thyrosphere size was calculated by measuring sphere areas using Image J software (NIH, Bethesda, MD, USA) (19).

To ascertain whether changes in thyrospheres’ size were due to proliferation rather than endoreplication, thyrospheres were dissociated into single cells by trypsin-EDTA treatment for 10 min with gentle pipetting and the number of viable cells counted.

Cell proliferation was also evaluated in cells exposed to metals in the presence of PD98059 (20 μM, Cell Signaling), a selective inhibitor of ERK1/2. The inhibitor was kept in the culture medium throughout the entire period of exposure to metals.

### Immunoblot Analyses

To measure the extracellular signal-regulated protein kinase (ERK1/2), thyrospheres were lysed and subjected to Western blot analysis, as previously described (20). The following antibodies against total and phosphorylated ERK1/2 were purchased from Cell Signaling Technology (Beverly, MA, USA): anti-ERK1/2, anti-P-ERK1/2 (T202/Y204) and anti-Vinculin. Bands were detected digitally using the Odyssey Fe imaging system (Li-COR Bioscience, Lincoln, NE, USA) and the blots were then quantified using the Li-COR Image Studio software version 5.2.5.

The metal concentration used for evaluating ERK1/2 activation at 5, 15 and 30 minute time points was selected on the basis of the peak effect of each metal on BrdU incorporation.

ERK phosphorylation was also measured in cells exposed to metals in the presence of PD98059 (20 μM) that was kept in the culture medium throughout the entire period of exposure to metals.

### Statistical Analysis

All data were expressed as mean ± SEM. Statistical analyses were performed using the GraphPad Prism 5.0 Software. All differences between mean values were evaluated by the Student’s *t*-test. A two-sided *P* < 0.05 was accepted as significant.

## Results

### Metal Effect on Thyroid Cell Proliferation

Parallel experiments carried out in both thyrospheres and differentiated thyrocytes indicated that all examined metals significantly increased BrdU incorporation in thyrospheres but not in thyrocytes.

**Figure 1:**
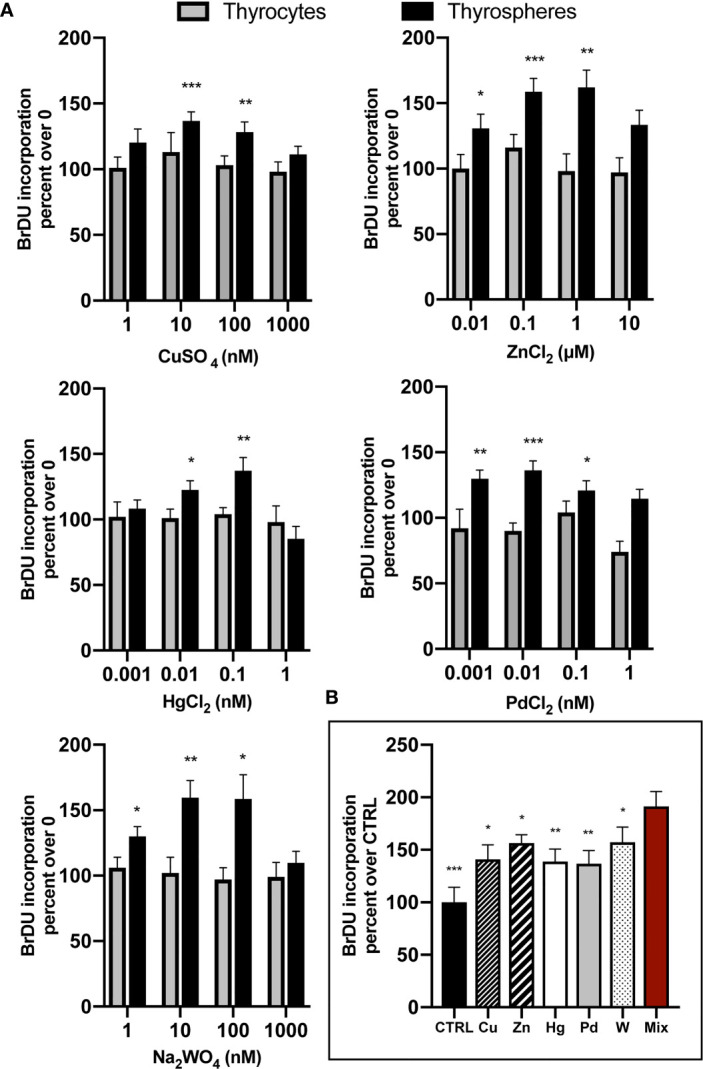
**(A)** Chronic exposure of human thyrospheres (aggregates of stem/precursor thyroid cells) to heavy metals at the indicated salt concentrations significantly increased BrdU incorporation in all cases. Basal values in untreated cells were always considered equal 100 and values of BrdU incorporation after cell exposure to metals were expressed as percent changes over basal. The dose-response curves followed in all cases a biphasic pattern, declining after the peak value when metal concentrations were further increased. Data shown for each metal indicate the average values ± SEM of four separate experiments except for W (ten separate experiments). In differentiated thyrocytes none of the five heavy metals studied or their mixture had any effect on BrdU incorporation. *p < 0.05, **p < 0.01 and ***p < 0.001 vs. 0. **(B)** The mixture of the five metals studied (Mix), each at the concentration causing the maximum BrdU incorporation, promoted BrdU incorporation significantly more than control thyrospheres (CTRL) and also significantly more than each metal acting alone (average values ± SEM of four separate experiments). *p < 0.05, **p < 0.01 and ***p < 0.001 vs. Mix.

Metals were investigated in a wide range of concentrations spanning 1,000-fold the lowest dose tested and always included the average metal concentration previously documented in the urine of residents of the volcanic area (μg/g creatinine assuming 1g of creatinine equivalent to 1 L of urine): Cu= 5.5, Zn= 217.0, W= 0.12, Hg= 0.21, Pd= 0.09 (14). In thyrospheres, BrdU incorporation was significantly increased after exposure to each metal examined, with peak values being significantly higher than basal values: Cu +36.7% ± 6.9, p<0.001; Zn +58.8% ± 10.1, p<0.001; W +59.5% ± 13.3, p<0.01; Hg +36.7% ± 10.1, p<0.01; and Pd +36.2% ± 7.2, p<0.001. Values then declined in all cases when the metal concentration was further increased (**Figure 1A**). In parallel experiments, thyrospheres were exposed to a mixture of the five examined metals, each at a concentration causing the greatest BrdU incorporation (Pd at 0.01 nM, Hg at 0.1 nM, Cu and W at 10 nM and Zn at 100 nM). The growth effect of this metal mixture on thyrospheres was +91.0% ± 14.8, which was significantly higher (p<0.001) than control thyrospheres and also significantly higher than the effect observed with each single component of the mixture acting alone at the same concentration (p<0.05 for Cu, W and Zn, p<0.01 for Hg and Pd, see **Figure 1B**)

No effect of the examined metals was observed in differentiated thyrocytes at any concentration tested (**Figure 1A**). Also the metal mixture had no effect (data not shown).

**Figure 2:**
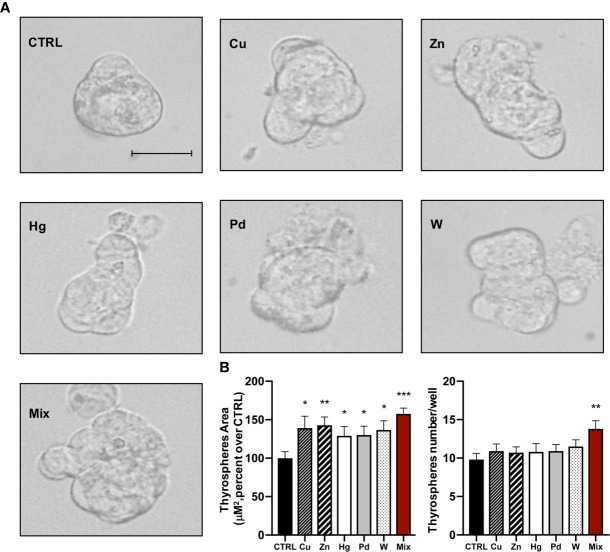
**(A)** Representative phase–contrast microscopy images of thyrospheres grown in standard medium (CTRL) or in medium added with each of the five heavy metals tested (at the salt concentration causing the maximum BrdU incorporation) or their mixture for 8 days (Scale bar: 30 μM). **(B)** Histograms indicate the mean value ± SEM of three separate experiments for measuring the size (μM^2^) and the number of thyrospheres after exposure to each metal or their mixture. *p < 0.05, **p < 0.01, ***p < 0.001 vs. CTRL.

To evaluate the growth effect caused by metals using an independent method, we measured the number and size of thyrospheres 8 days after exposure to each metal at the concentration causing the maximum effect on BrdU incorporation and to the metal mixture. All metals caused an increase in the thyrosphere size and also an altered morphology (**Figure 2A**). Average size values indicated that the increase (μM^2^) was significant for all metals (p<0.05 for all metals except zinc p<0.01; **Figure 2B**). The metal-induced growth effect observed with this procedure roughly reflected the effect observed with the BrdU incorporation method, with the greatest effect caused by thyrosphere exposure to the metal mixture. In contrast, chronic exposure to metals did not increase the number of thyrospheres. Only the metal mixture had such an effect, significantly increasing the number of thyrospheres (p<0.01) relative to both control thyrospheres and also thyrospheres exposed to each individual metal (**Figure 2B**).

Counting viable cells of dissociated thyrospheres indicated that proliferation rather than endoreplication was involved in the effect of metals on thyrospheres.

### Metal Effect on ERK1/2 Phosphorylation

To investigate the mechanism involved in the metal-induced growth in human thyrospheres, we measured the extracellular signal-regulated protein kinase phosphorylation (ERK1/2), a pathway that is already reported to be activated by cell exposure to metals (17, 21–24).

**Figure 3:**
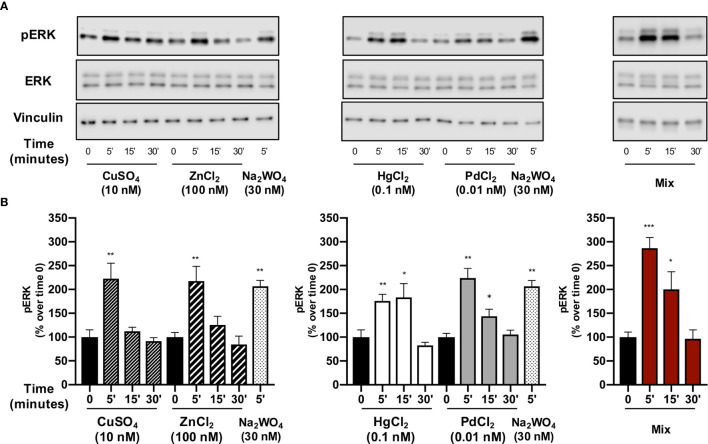
The effect of metals on ERK1/2 phosphorylation in thyrospheres. Thyrospheres were treated for 30 min with the indicated metals at the concentrations causing maximum BrdU incorporation, lysed at the indicated times and analyzed for ERK1/2 phosphorylation by Western blot. **(A)** Representative immunoblots from six separate experiments. **(B)** Histograms represent the mean value ± SEM of densitometric values normalized to vinculin and expressed as percent of time 0. *p < 0.05, **p < 0.01, ***p < 0.001.

Each single metal rapidly and significantly (p<0.01) stimulated ERK1/2 phosphorylation with an increase that was over 100% for all metals except Hg. The peak value occurred at 5 min and then values decreased, returning to basal levels after 30 min. Hg had a more prolonged effect, but ERK1/2 phosphorylation returned to basal values at 30 min also with this metal. Again, thyrosphere exposure to the metal mixture caused ERK1/2 phosphorylation that was significantly greater than that observed with each single metal alone (**Figure 3**).

Thyrospheres pre-treatment with PD98059 (30 min) decreased the ERK phosphorylation induced by the exposure (5 min) to each single metal and their mixture (**Supplemental Figure 1**).

### Effect of ERK1/2 Inhibition on Metal-Induced Cell Proliferation and Secondary Thyrospheres’ Formation

**Figure 4:**
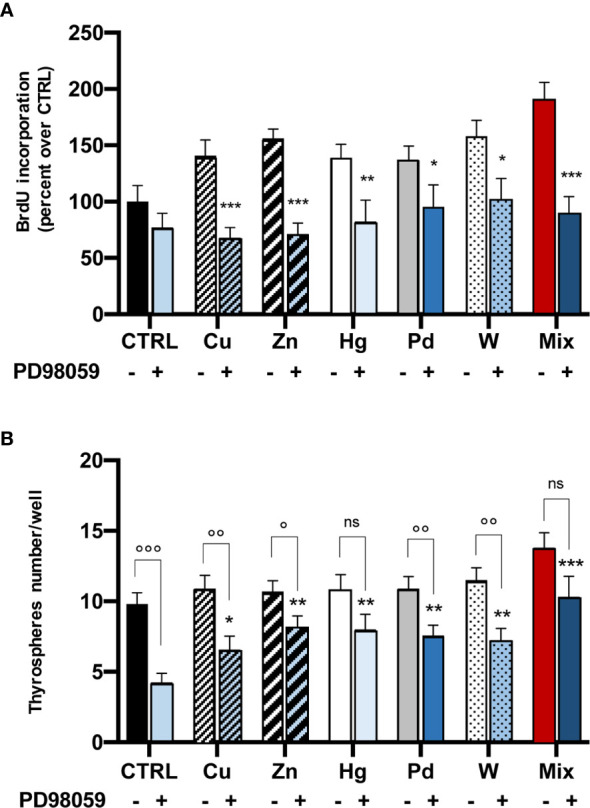
Effect of the ERK1/2 inhibitor PD98059 on metal-induced BrdU incorporation (panel A, cell proliferation) and secondary thyrospheres’ formation (panel B). **(A)** Histograms represent the mean value ± SEM of three separate experiments. For each metal and their mixture values are expressed as percent of CTRL (untreated thyrospheres). After thyrospheres’ exposure to metals for 3 days in the absence or the presence of PD98059 (20 µM) BrdU incorporation was significantly iblunted in all cases by the inhibition of the ERK1/2 pathway. *p < 0.05; **p < 0.01 and ***p < 0.001 vs. CTRL. **(B)** Inhibition of the ERK1/2 pathway reduces secondary thyrospheres’ formation. Cells from dissociated thyrospheres were plated at a density of 3,000 cells in nonadherent 96-wel plates and exposed to metals in the absence or the presence of PD98059 (20 µM) for 8 days. Spheres were counted in 4 wells for each condition. The results are expressed as mean value ± SEM of three separate experiments. *p < 0.05; **p < 0.01 and ***p < 0.001 comparing thyrospheres’ number in the presence of PD98059 to the number measured under the same condition in control thyrospheres. ns, non significant; °p < 0.05; °°p < 0.01 and °°°p < 0.001 comparing thyrospheres’ number in the absence or the presence of PD98059.

To confirm that the ERK1/2 signaling pathway plays a major role in thyrosphere growth after exposure to metals, we measured BrdU incorporation after exposure to metals in the presence of the ERK1/2 inhibitor PD98059. In this condition, thyrospheres proliferation was significantly reduced for all metals, with a different effect for different metals: the ERK1/2 inhibition was greater for Cu, Zn and for the metal mixture (p<0.001), was less significant for Hg (p<0.01) and smaller for Pd and W (p<0.05) (**Figure 4A**).

Inhibition of the ERK1/2 pathway also affected the thyroid stem cell self-renewal, reducing the number of secondary thyrospheres. No significant change was observed in thyrospheres’ size or morphology. The decrease in thyrosphere number was greater in control (not metal-exposed) thyrospheres (p<0.001 in comparison with thyrospheres grown in the absence of the inhibitor). The PD98059 effect on secondary thyrosphere number was present to a lesser extent for all metals (range -23 to -40%), and was significantly lower than observed in control thyrospheres (**Figure 4B**). It should be noted that in thyrospheres exposed to Hg or to the metal mixture, the number was reduced relative to thyrospheres cultivated in the absence of PD98059, but the difference was not statistically significant (**Figure 4B**).

## Discussion

Using both differentiated and undifferentiated human thyroid cells in primary culture, the present study demonstrates that five heavy metals (Cu, Hg, Pd, W and Zn) promote proliferation in thyroid stem/precursor cells, but not in differentiated thyrocytes. This effect occurs after chronic cell exposure to metal concentrations in the same range observed in the volcanic environment. The study also demonstrates that metal-induced proliferation occurs *via* activation of the ERK1/2 pathway and that the mixture of the five metals has a significantly greater effect than each single metal acting alone.

Exposure to the studied metals increased BrdU incorporation in thyrospheres with a maximum growth increase ranging from +35% to +59% for the different metals. This peak effect was reached at metal concentrations in the nanomolar range, similar to those measured in the urine of residents from the volcanic area in Sicily, where the thyroid cancer incidence is doubled in comparison to adjacent non-volcanic areas (7). For all metals, the effect on BrdU incorporation followed a biphasic pattern, decreasing after the peak value when the metal concentration was further increased. This bimodal dose-response of a biological effect is typical of hormesis, a well-recognized phenomenon occurring at very low concentrations of the stimulating agent and observed both *in vitro* and *in vivo* (15, 25). This chemically-induced hormesis occurs for many compounds and trace elements, including heavy metals (26), and can be cell-specific (27), life-stage specific (28) and also depend on the duration of exposure (29).

We now observe that a hormesis-driven growth response is observed in immature but not in well-differentiated human thyroid cells after chronic exposure to nanomolar concentrations of Cu, Hg, Pd, W and Zn. The different response is certainly based on the different genetic and biological characteristics of cells at different stages of differentiation. In translational terms, this observation highlights the increased sensitivity of thyroid cells to environmental heavy metals during fetal life and also of stem cells present in the adult thyroid. Immature thyroid cells, therefore, may suffer cytotoxic damage from metal concentrations that are not unhealthy for well-differentiated thyrocytes. If progenitors exposed to such a low dose of metals produce a progeny of mature thyroid cells prone to transformation (17, 30–32), this process could favor an increased incidence of thyroid cancer in the population.

Morphological evidence indicates that this growth effect concerns the size rather than the number of thyrospheres (except for the metal mixture) suggesting that exposure to metals primarily affects the proliferating capacity of thyroid precursor cells rather than the self-renewal of thyroid stem cells. Moreover, thyrospheres exposed to slightly increased metals show an abnormal shape, suggesting that the orderly cell aggregation in spheres is also affected. The mechanisms and consequences of this phenomenon require additional studies.

An important observation is that the mixture of the five metals, each at a fixed concentration determined by its maximal effect on stimulating proliferation, has a significantly greater effect than each single metal used alone at the same concentration used in the mixture. The more potent effect of the mixture can be the final result of different mechanisms concerning the synergistic and antagonistic relationship between different metals that can interfere in the uptake, accumulation and interaction of other metals with cellular biological mechanisms (33). Our data concern a simplified and limited chemical mixture: actually each component of the mixture, at a different concentration than used in our study, could differently interact with the other components determining different thresholds for their biological effects (34, 35). For this reason, our data on the metal mixture are of limited general significance. They are sufficient, however, to highlight the complexity of defining the effect of a multiple and variable environmental pollution on the cell biology, including the transformation and carcinogenic potential. As a consequence, it will be difficult for government agencies to assess the public health risk when dealing with the exposure to multiple chemicals, even when they are increased at a low-level (36).

The ERK1/2 signaling pathway is a major effector of metal-induced proliferation. This is an already reported mechanism, but we observe it at nanomolar metal concentrations, much lower than usually tested (21, 23, 24). Quantitatively, the effect of metals on the ERK1/2 activation was similar for all compounds examined, but this does not necessarily imply similar activation mechanisms. The relative potency of each metal may be the final result of different biological effects, possibly combined, and including the possible generation of reactive oxygen species, the inhibition of phosphatases and also different metal-specific mechanisms, such as the activation of zinc-sensing receptors for the Zn effect (22).

A major role of the ERK1/2 signaling pathway in the metal-induced growth of thyrospheres is confirmed by the significant decrease in BrdU incorporation when metal stimulation occurred in the presence of the specific ERK1/2 inhibitor PD98059 (**Figure 4**). The inhibition of ERK1/2 signaling significantly reduced the number of secondary thyrospheres, an effect that was mostly evident in control thyrospheres (not exposed to metals), suggesting that this pathway is relevant for immature thyrocyte self-renewal and proliferation. The metal-induced growth of thyrospheres, however, was only partially inhibited by the presence of PD98059 (**Figure 4**), suggesting that the ERK1/2 pathway is indeed involved, but that other unexplored mechanisms may be activated. In addition to ERK, in fact, other mitogen activated protein kinases (MAPK), such as c-Jun NH_2_-terminal kinase (JNK) and p38 MAPK, may be activated by extracellular signals and regulate cell functions like growth, differentiation and apoptosis. All of these pathways could respond to changes in the cellular environment, like the increase of heavy metals (37), and initiate the downstream induction of transcription factors such as the Nuclear Factor-kappa B (NF-kb) which, in turn, may mediate a variety of cell processes (38). In this complex network, most data indicate that ERK is generally activated by mitogenic stimuli (as in our study), while both JNK and p38 are more involved in the regulation of apoptosis (39, 40). This evidence, however, is not univocal, as it has always been observed with metal concentrations in the micromolar range (2-3 orders of magnitude greater than in our study), and may be metal and cell-type specific (37).

In conclusion, some potentially toxic heavy metals can stimulate the growth of thyroid stem/precursor cells at environmentally relevant concentrations that have no effect on mature thyrocytes. Life-long (including pre-natal and early life) exposure to such a mixture of slightly increased metals may affect undifferentiated thyroid cells, making them (and possibly their progeny) more susceptible to future damaging factors. Further studies are required to assess the relevance of this phenomenon on the increased incidence of thyroid cancer.

## Data Availability Statement

The original contributions presented in the study are included in the article/**Supplementary Material**. Further inquiries can be directed to the corresponding author.

## Ethics Statement

The studies involving human participants were reviewed and approved by Ethics committee “Catania 2”, ARNAS Garibaldi, Catania, P.zza S. M. di Gesù n. 5. The patients/participants provided their written informed consent to participate in this study.

## Author Contributions

FG and RV conceived the study and planned the experiments. FG, RM, and GP performed the *in vitro* experiments. MT and AF selected and operated the patients that provided informed consent to donate thyroid tissue for the *in vitro* experiments. RV and FG prepared the manuscript and FG and RM prepared the figures. All authors contributed to the article and approved the submitted version.

## Funding

These studies have received funding from AIRC (Fondazione AIRC per la Ricerca sul Cancro, Italy) under IG 2017 – ID. 19897 – P.I. RV.

## Conflict of Interest

The authors declare that the research was conducted in the absence of any commercial or financial relationships that could be construed as a potential conflict of interest.

## Supplementary Material

Supplementary Figure 1 Pre-treatment with PD98059 inhibits ERK1/2 phosphorylation induced by metals in thyrospheres. **(A)** Thyrospheres pre-treated or not with PD98059 (20 μM for 30 min) were exposed to the studied metals or their mixture for 5 min. Western blot analysis was then carried out in thyrospheres’ whole lysates to evaluate ERK1/2 phosphorylation. A representative immunoblot from three independent experiments is shown. **(B)** Histograms represent the mean ± SEM of densitometric values normalized to vinculin and expressed as percent of control (not treated) thyrospheres. *p < 0.05; **p < 0.01 and ***p <0.001 comparing ERK1/2 phosphorylation in the absence or the presence of PD98059.
