# Supplementary figures and images for: Thyroid Stem Cells But Not Differentiated Thyrocytes Are Sensitive to Slightly Increased Concentrations of Heavy Metals
Source: Front Endocrinol (Lausanne). 2021 Apr 19;12:652675. doi: 10.3389/fendo.2021.652675 (PMC8092438; doi:10.3389/fendo.2021.652675)

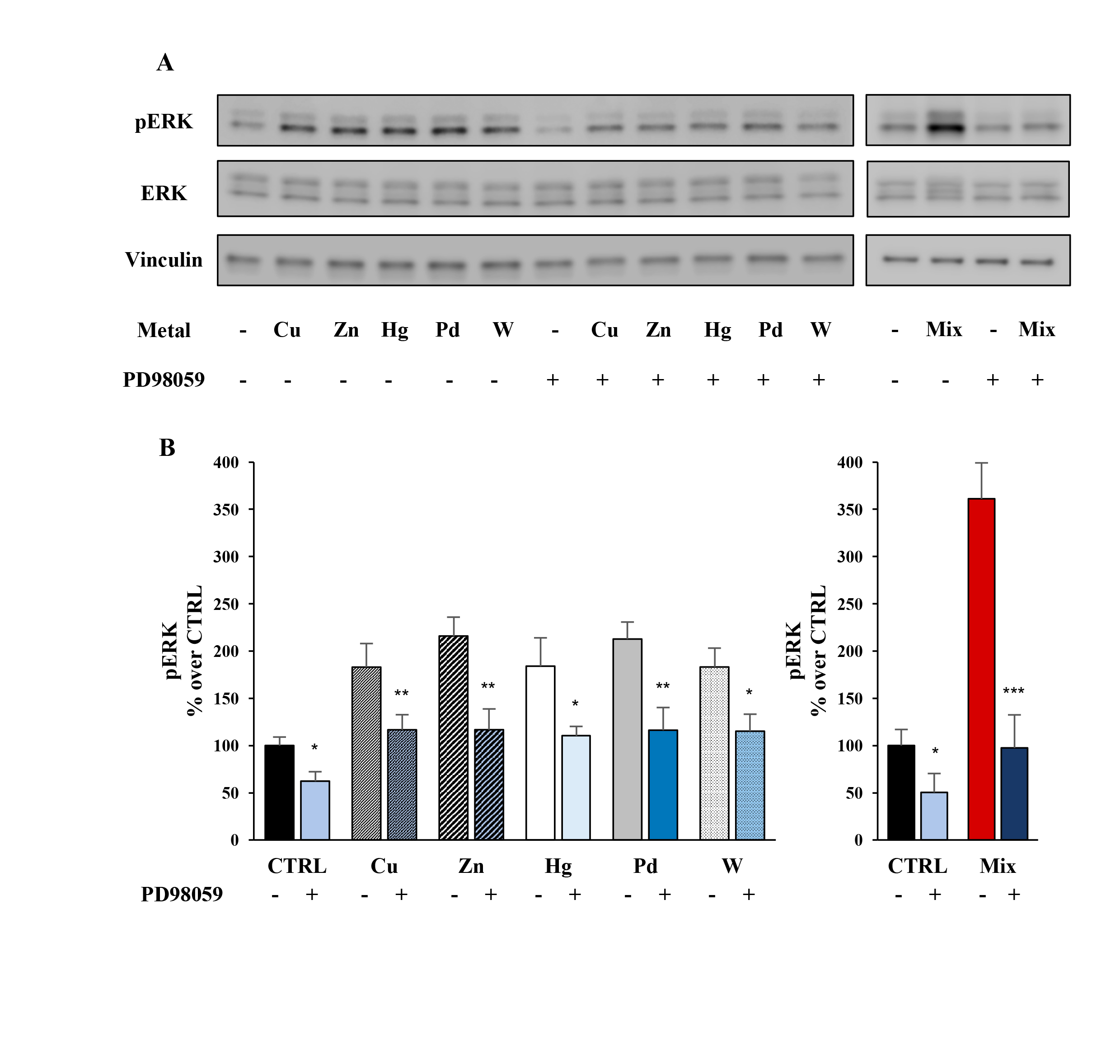

Supplement: Supplementary Figure 1 — Pre-treatment with PD98059 inhibits ERK1/2 phosphorylation induced by metals in thyrospheres. (A) Thyrospheres pre-treated or not with PD98059 (20 μM for 30 min) were exposed to the studied metals or their mixture for 5 min. Western blot analysis was then carried out in thyrospheres’ whole lysates to evaluate ERK1/2 phosphorylation. A representative immunoblot from three independent experiments is shown. (B) Histograms represent the mean ± SEM of densitometric values normalized to vinculin and expressed as percent of control (not treated) thyrospheres. *p < 0.05; **p < 0.01 and ***p <0.001 comparing ERK1/2 phosphorylation in the absence or the presence of PD98059. [file Image_1.jpeg]
